# The impact of perceived everyday discrimination and income on racial and ethnic disparities in PTSD, depression, and anxiety among veterans

**DOI:** 10.1371/journal.pone.0291965

**Published:** 2023-09-26

**Authors:** Yael I. Nillni, Arielle Horenstein, Juliette McClendon, Christopher C. Duke, Molly Sawdy, Tara E. Galovski

**Affiliations:** 1 National Center for PTSD, Women’s Health Sciences Division at VA Boston Healthcare System, Boston, MA, United States of America; 2 Department of Psychiatry, Boston University Chobanian & Avedisian School of Medicine, Boston, MA, United States of America; 3 VA Boston Healthcare System, Boston, MA, United States of America; 4 Altarum, Ann Arbor, MI, United States of America; 5 Suffolk University, Boston, MA, United States of America; The University of Alabama, UNITED STATES

## Abstract

**Objectives:**

Black and Hispanic/Latinx individuals experience a greater burden of mental health symptoms as compared to White individuals in the general population. Examination of ethnoracial disparities and mechanisms explaining these disparities among veterans is still in its nascence. The current study examined perceived everyday discrimination and income as parallel mediators of the association between race/ethnicity and PTSD, depression, and general anxiety symptoms in a sample of White, Black, and Hispanic/Latinx veterans stratified by gender.

**Methods:**

A random sample of 3,060 veterans living across the U.S. (oversampled for veterans living in high crime communities) completed a mail-based survey. Veterans completed self-report measures of perceived discrimination via the Everyday Discrimination Scale, PTSD symptoms via the Posttraumatic Stress Disorder Checklist-5, depressive symptoms via the Patient Health Questionnaire, and anxiety symptoms via the Generalized Anxiety Disorder Questionnaire.

**Results:**

Models comparing Black vs. White veterans found that the significant effect of race on PTSD, depression, and anxiety symptoms was mediated by both perceived discrimination and income for both male and female veterans. Results were less consistent in models comparing Hispanic/Latinx vs. White veterans. Income, but not perceived discrimination, mediated the relationship between ethnicity/race and depression and anxiety symptoms, but only among women.

**Conclusions:**

Results suggest that discrimination and socioeconomic status are important mechanisms through which marginalized social status negatively impacts mental health.

## Introduction

Approximately 25% of veterans identify as a racial or ethnic minority, numbers which are projected to grow to almost 40% in the next three decades [1]. As the diversity of the veteran population continues to grow, attention to disparities in health and the mechanisms explaining these disparities is crucial to ensuring high quality and equitable care for all veterans.

Posttraumatic stress disorder (PTSD), major depressive disorder (MDD), and anxiety-related disorders are common mental health conditions experienced by veterans of the United States military, with current prevalence estimates for PTSD, MDD, and anxiety disorders ranging from 4.8–23% [2–4], 9.6% [5], and 3.7–4.8% [6, 7], respectively. There is growing evidence of ethnoracial disparities in PTSD, MDD, and anxiety-related disorders among veterans. Black and Hispanic/Latinx veterans have increased risk for PTSD [4, 8–10], a higher prevalence, incidence, and severity of PTSD after exposure to trauma [11–15], and experience a more severe course of illness of PTSD [16, 17] compared to non-Hispanic/Latinx White veterans (hereafter referred to as White).

There is less research regarding ethnoracial disparities in depression among veterans. In the general population, Non-Hispanic/Latinx Black (hereafter referred to as Black) and Hispanic/Latinx individuals have been found to have higher rates of depression compared to White individuals [18–20] and are vulnerable to more persistent mood disorders over the lifetime [21]. However, in a study comparing depression prevalence in Black and White veterans over a 12-year period (2005–2016), the opposite was found with White veterans having higher rates of depression than Black and Hispanic/Latinx veterans during each year examined [5].

Findings on ethnoracial disparities in anxiety are also somewhat mixed in both civilian and veteran populations. Within the general population, White individuals have been found to have a higher 12-month and lifetime prevalence of anxiety disorders compared to Black, Asian, and Hispanic/Latinx individuals [22, 23]. However, Black and Hispanic/Latinx individuals experience a greater persistence of anxiety disorders across the lifetime [21]. Findings are similarly mixed among the veteran population, with one study finding that the majority of ethnoracial minoritized veterans report lower rates of anxiety disorders compared to White veterans [24], but another study finding that among female veterans, ethnoracial minorities report greater anxiety symptoms compared to their White counterparts [14]. This latter finding points to the importance of understanding the intersection of race and gender in examinations of mental health disparities in order to have a more precise understanding of which subgroups in the veteran population are most vulnerable to mental health symptoms.

Minority stress theory, which in part posits that social and structural processes, including discrimination, are important mechanisms through which marginalized social status negatively impacts mental health [25] may explain some of the ethnoracial disparities in mental health. Furthermore, there is unequal distribution of wealth across racial and ethnic groups in the United States [26], which has been consistently linked to ethnoracial disparities in mental health [18, 27, 28]. Specifically, low income has been associated with increased odds of having an anxiety disorder [29, 30], depression [31], as well as PTSD prevalence [32]. Furthermore, lower socioeconomic status accounts for higher rates of depression in Black and Hispanic/Latinx adolescents compared to White adolescents [33] and financial distress (i.e., worries about and/or difficulty meeting expenses and bills) is more strongly associated with depression for Black individuals compared to White individuals [34].

The experience of systemic and chronic racism and discrimination by minority and marginalized groups has also been linked to poorer mental health outcomes [35]. Studies in civilian populations have indicated that perceived discrimination is associated with the presence and chronicity of depression [36–38] and PTSD [39–41], as well as multiple anxiety-related conditions including generalized anxiety disorder [42, 43], social anxiety disorder [44], panic disorder [45], and diffuse anxiety symptoms [46, 47].

Although a large body of research documents both the negative impact of discriminatory experiences and lower access to economic resources on the mental and physical health of racial/ethnic minorities, relatively few studies have examined the extent to which discrimination and economic resources explain ethnoracial disparities in mental health of veterans. One study found that both discriminatory stress and PTSD severity were higher in Black and Hispanic/Latinx Veterans compared to White Veterans, and that discriminatory stress was associated with increased PTSD severity over time [48]. This study highlights discrimination as an important psychosocial stressor that may contribute to ethnoracial disparities in veterans’ mental health. Socioeconomic markers such as education and income have also been found to account for the likelihood of a positive PTSD screen or onset among ethnoracial minoritized veterans compared to White veterans [8, 10].

Examination of mental health disparities often focus on single categories of identity (e.g., race or gender). However, such approaches do not reflect the complexity of people’s identities and the fact that many individuals have multiple and intersecting identities that interact to define each person’s unique experience [49]. Furthermore, individuals at the intersection of multiple marginalized identities (e.g., Black women) may be vulnerable to unique experiences of discrimination and inequity that cannot be explained by race or gender alone. Given that these unique experiences may compound discriminatory stress [50] and lead to even poorer mental and physical health outcomes [51, 52], it is important to examine mental health disparities through an intersectional lens. For example, research has found a stronger association between discriminatory stress and mental health outcomes (e.g., PTSD severity) among female veterans compared to male veterans, but only among specific ethnoracial groups [48]. Examining intersectional disparities in mental health outcomes among veterans is key to the accurate identification of subgroups of veterans at highest risk for poorer mental health outcomes and the psychosocial stressors that contribute to them.

In the current study we examined both perceived discrimination from multiple potential sources and income as parallel mediators of the relationship between race/ethnicity and PTSD, depression, and general anxiety symptoms in a sample of White, Black, and Hispanic/Latinx veterans. To further understand differences between race/ethnicity within gender, analytic models were stratified by gender.

## Methods

### Participants

Data for this study comes from the Longitudinal Investigation of Gender, Health, and Trauma (LIGHT) study, a prospective study examining the impact of exposure to ongoing trauma and violence on veterans’ mental health, functioning, and reproductive health. In order to address the primary aims of the larger investigation, female veterans and veterans living in high crime communities were oversampled. The VA/DoD Identity Repository (VADIR), a VA-managed dataset of all separated service members, was used to identify eligible veterans. High crime communities (i.e., overall crime index of at least 2 times higher than then national average) were determined by zipcode using ArcGIS. A random sample of veterans between the ages of 18 and 50 were selected such that 19,441 (69.4%) veterans were living within high crime zipcodes and 8,559 (30.6%) veterans were living within not high crime zip codes (i.e., all other zip codes). Female and male veterans were sampled in equal numbers from both zipcode groups. A total of 17,178 veterans were invited to participate [10,377 (60.4%) from high crime communities and 6,801 (39.6%) from not high crime communities] after accounting for duplicate and non-deliverable addresses (n = 10,822, 39%). Of those invited, 3,544 veterans enrolled in the study (21% response rate), which included 2,047 (57.8%) veterans residing in high crime communities and 1,838 (51.9%) women. Veterans living in not high crime communities (22.0% vs. 19.7%), rural areas (21.5% vs. 20.4%), and/or were female (24.5% vs. 17.6%) were more likely to enroll in the study. For the current analysis, a total of 3,060 veterans who identified as Black, White, and/or Hispanic/Latinx were included.

### Procedures

Data was collected via a mail-based survey administered using a modified Dillman mail survey approach [53]. Veterans living in the United States first received an invitation letter, study fact sheet, survey, an opt out postcard, and a $5 cash pre-incentive [54]. One and a half weeks following the initial invitation, veterans received a reminder post-card, and one and a half weeks following the postcard they received a second reminder letter and survey. Veterans who completed the survey received an additional $20. All procedures were approved by the VA Boston Healthcare Institutional Review Board. Veterans provided informed consent for this study.

### Measures

#### Race/Ethnicity

Race and ethnicity were ascertained using the clinical research reporting policies outlined by the National Institutes of Health [1], which defines ethnicity as Hispanic/Latinx vs. Not Hispanic/Latinx and five racial categories (i.e., American Indian or Alaska Native, Asian, Black or African American, Native Hawaiian or Other Pacific Islander, and White). There were low base rates in our sample of Non-Hispanic Native American (1.0%) Asian (2.5%), Middle Eastern/North African (0.1%), Native Hawaiian (0.4%), Pacific Islander (0.7%), and Other/Multiracial (7.7%). Therefore, only veterans who reported being Hispanic/Latinx, Black, and/or White were included in these analyses to create three groups: Non-Hispanic/Latinx White (i.e., White), Non-Hispanic/Latinx Black (i.e., Black), and Hispanic/Latinx. Veterans reporting Hispanic/Latinx origin were grouped as Hispanic/Latinx regardless of race [46.9% identified as White and no other racial group; 11.0% identified as White and another racial group (36.1% Native American, 10.9% Black, 4.3% Asian, 4.3% Middle Eastern/North African, 23.9% other, and 28.3% as Multiracial), and 36.9% identified as a non-White race (7.1% Native American, 12.3% Black, 5.8% Asian, 0.6% Middle Eastern/North African, 59.4% Other, and 12.3% Multiracial).

#### Demographic and military characteristics

Household income was self-reported by asking the veteran to estimate their household’s yearly gross income. They were asked to include all sources of income from all earners in their household and to choose an income range from “no income” to “$150,000 or more per year” (i.e., no income, less than $15,000 per year, $15,000 –$24,999, $25,000 - $34,999, $35,000 - $44,999, $45,000 - $54,999, $55,000 - $74,999, $75,000 - $99,999, $100,000 - $149,000). Other demographic information, including gender, educational attainment, rank, and deployment status were also collected via self-reported.

#### Perceived everyday discrimination

The Everyday Discrimination Scale (EDS) [55] is a widely used 9-item measure that assesses individuals’ experiences of everyday discrimination (i.e., “you are treated with less courtesy than other people are”) on a 6-point Likert scale from 0 (*never*) to 5 (*almost every day*) with scores ranging from 0–45. Participants also report what they believe are the main reasons for these experiences (i.e., ancestry or national origins, gender, race, age, religion, height, weight, some other aspect of physical appearance, sexual orientation). The EDS has shown good internal consistency and test-retest reliability [56]. Internal consistency reliability in the current sample was excellent (*α* = .91).

#### PTSD

The Posttraumatic Stress Disorder Checklist-5 (PCL-5) [57] is a 20-item measure that assesses the severity of each of the Diagnostic and Statistical Manual of Mental Disorders (DSM-5) PTSD symptoms anchored to the worst event. To determine the worst event participants were asked to “*think about things that may have happened to you throughout your life that are unusually or especially frightening*, *horrible*, *or traumatic*. *If you have had one of these experiences*, *which experience causes you the most distress*?” Then, they noted which type of event it was (combat/exposure to warzone, physical assault, sexual assault, accident, natural disaster, seen someone killed or seriously injured, death of loved one through homicide or suicide, or other). Time since trauma was on average 15.4 years (SD = 10.1) with little variation between race/gender groups. Participants then rated the extent to which each PTSD symptom (e.g., “repeated, disturbing, and unwanted memories of the stressful experience”) bothers them in the past month from 0 (*not at all*) to 4 (*extremely*) with scores ranging from 0–80. The PCL-5 has demonstrated sound psychometric properties among both civilians [57] and veterans [58] with a cut-off score of ≥ 33 indicating probable PTSD diagnosis. Among trauma-exposed veterans receiving care at the VA, the mean PCL-5 score has been found to be 36.97 (SD = 21.16) [58]. Internal consistency reliability in the current sample was excellent (*α* = .97).

#### Depression

The Patient Health Questionnaire (PHQ-9) [59] is a 9-item measure that assesses each of the DSM-5 depression symptoms (e.g., “feeling down, depressed, or hopeless) in the past two weeks from 0 (*not at all*) to 3 (*nearly every day*) with scores ranging from 0–27 and scores of ≥ 10 indicating moderate to severe depressive symptoms. The PHQ-9 has demonstrated excellent internal reliability, test-retest reliability, and strong criterion and construct validity [59]. Internal consistency reliability in the current sample was excellent (*α* = .93).

#### Anxiety

The Generalized Anxiety Disorder Questionnaire (GAD-7) [60] is a 7-item measure that screens for generalized anxiety disorder. Participants rate the extent to which they experience each symptom (e.g., “feeling nervous, anxious, or on edge”) in the past two weeks from 0 (*not at all*) to 3 (*nearly every day*) with scores ranging from 0–21 and scores of ≥ 10 indicating moderate to severe anxiety symptoms. The GAD-7 has demonstrated excellent validity, internal consistency, and test-retest reliability [60]. Internal consistency reliability in the current sample was excellent (*α* = .95).

### Data analytic plan

Analyses were conducted using SPSS version 24 and PROCESS version 3.5 [61]. Parallel mediation models were tested with PROCESS in SPSS using race/ethnicity as the independent variable, EDS and income as parallel mediators, age and education as covariates, and mental health measures (i.e., PHQ-9, GAD-7, and PCL-5) as the outcome. In order to examine race differences within gender, models were stratified by race/ethnicity and gender (i.e., Black vs. White comparison among males; Black vs. White comparisons among females; Hispanic/Latinx vs. White comparisons among males; Hispanic/Latinx vs. White comparisons among females) for each of the three mental health outcomes, totaling 12 models. Given the small number of Veterans identifying as transgender (n = 9) or other (n = 12), comparisons between racial/ethnic groups within this gender group were not possible. Therefore, only veterans identifying as male or female were included in the analysis. Bootstrapping with 95% CI was used to determine mediation significance. Bootstrapping is preferred over alternatives such as a Sobel test because of more flexible statistical assumptions, greater statistical power, and lower Type I error rate [61]. Given that mediation analyses were conducted on cross sectional data, competing models examining mental health symptoms as the mediator were conducted as a sensitivity analysis.

## Results

### Differences in demographic and study variables across race/ethnicity groups

Full sample demographics are presented in Table 1. The sample was 50.29% (*n* = 1,539) female and mostly ranged in age between 34 to 49-years-old (57.45%; *n* = 1,758). Participants were 62% (*n* = 1,906) White, 24% (*n* = 734) Black, and 13% (*n* = 420) Hispanic/Latinx. As compared to White veterans, Black veterans were more likely to be older and female and were less likely to have been deployed. Hispanic/Latinx veterans were more likely to be younger than both White and Black veterans, and more likely to be male as compared to Black veterans. White veterans were the most likely to have a college degree and higher income, followed by Hispanic/Latinx veterans and then Black veterans.

**Table 1:** Demographic differences by race/ethnicity group.

	White (non-Hispanic/Latinx)		Black (non-Hispanic/Latinx)			Hispanic/Latinx (any race)			
	n = 1906		n = 734			n = 420			
Variables	n	%	n	%	v^1^	n	%	v^1^	v^2^
**Age**					.097***			.059*	.171***
18–34	775	41.16%	222	31.01%		192	46.27%		
34–49	1068	56.72%	469	65.50%		221	53.25%		
50+	40	2.12%	25	3.49%		2	0.48%		
**Gender**					.116***			0.028	.090**
Male	978	52.36%	284	39.44%		202	48.67%		
Female	890	47.64%	436	60.56%		213	51.33%		
**Branch**					.145***			.094***	.148***
Army	858	45.13%	428	58.47%		220	52.51%		
Marine Corps	180	9.47%	40	5.46%		55	13.13%		
Navy	342	17.99%	136	18.58%		68	16.23%		
Air Force	476	25.04%	125	17.08%		70	16.71%		
Coast Guard	45	2.37%	3	0.41%		6	1.43%		
**Mil Occupation**					.138***			.024	.126***
Combat arms	389	21.28%	83	11.99%		75	18.75%		
Combat support	665	36.38%	219	31.65%		148	37.00%		
Service support	774	42.34%	390	56.36%		177	44.25%		
**Deployed**	1175	63.14%	373	52.83%	.094***	236	58.85%	.033	.058
**Education**					.162***			.075**	.094**
High School	134	7.14%	91	12.59%		32	7.80%		
Some College	738	39.34%	371	51.31%		198	48.29%		
College +	1004	53.52%	261	36.10%		180	43.90%		
**Income**					.329***			.117***	.256***
No income	15	0.81%	25	3.54%		6	1.48%		
Less than $15k	93	5.00%	75	10.61%		30	7.41%		
$15k - $24.9k	114	6.13%	111	15.70%		45	11.11%		
$25k - $34.9k	148	7.96%	108	15.28%		37	9.14%		
$35k - $44.9k	152	8.17%	99	14.00%		32	7.90%		
$45k - $54.9k	179	9.62%	76	10.75%		38	9.38%		
$55k - $74.9k	280	15.05%	92	13.01%		72	17.78%		
$75k - $99.9k	313	16.83%	63	8.91%		58	14.32%		
$100k - $149.9k	323	17.37%	41	5.80%		56	13.83%		
$150k+	243	13.06%	17	2.40%		31	7.65%		
**Meets Clinical Cutoff**									
PHQ-9 Clin Cutoff	478	26.51%	277	40.44%	.135***	132	33.42%	.059**	.070*
GAD-7 Clin Cutoff	435	23.29%	256	36.73%	.134***	108	26.67%	.029	.103***
PCL-5 Clin Cutoff	401	21.88%	238	35.36%	.137***	110	27.85%	.054*	.077*
**Type of Trauma**					.105***			.055	.084
Combat/warzone	243	14.96%	66	11.96%		41	10.78%		
Physical assault	89	5.48%	38	6.88%		21	6.29%		
Sexual assault	219	13.49%	94	17.03%		58	17.37%		
Accident	82	5.05%	30	5.43%		16	4.79%		
Natural disaster	19	1.17%	10	1.81%		4	1.20%		
Saw death/injury	88	5.42%	27	4.89%		19	5.69%		
Homicide/Suicide	89	5.48%	53	9.60%		20	5.99%		
Other	162	9.98%	44	7.97%		36	10.78%		
None	633	38.98%	190	34.42%		119	35.63%		
	mean	SD	mean	SD	d^1^	mean	SD	d^1^	d^2^
EDS	9.49	8.67	12.48	10.55	0.310***	10.86	9.69	0.149**	-0.160*
PHQ-9	6.59	6.56	8.94	7.89	0.324***	7.96	7.09	0.201***	-0.131*
GAD-7	5.74	5.97	7.62	6.93	0.291***	6.79	6.27	0.172**	-0.126*
PCL-5	24.48	21.62	32.95	23.96	0.371***	27.98	23.02	0.157*	-0.212**

*Note*. v1 = Cramer’s V compared to White; v2 = Cramer’s V compared to Black; d1 = Cohen’s d compared to White; d2 = Cohen’s d compared to Black; EDS = Everyday Discrimination Scale; GAD-7 = Generalized Anxiety Disorder Questionnaire; PCL-5 = Posttraumatic Stress Disorder Checklist for DSM-5; PHQ-9 = Patient Health Questionnaire

Not all counts sum to the total N due to missing data on individual survey questions; clinical cut off proportions on the PCL-5 are out of the entire sample with available data.

* *p* < .05

** *p* < .01

*** *p* < .001

EDS scores were significantly higher for both Black veterans (*M* = 12.48) and Hispanic/Latinx veterans (*M* = 10.86) compared to White veterans (*M* = 9.49). Moreover, Black veterans had a significantly higher EDS mean score compared to Hispanic/Latinx veterans. Of the 10 potential reasons for perceived discrimination, the most frequently rated reason for discrimination for Black male veterans was race (64.75%) followed by gender (24.46%). For Black female veterans, it was race (65.06%) followed by gender (53.10%). The most frequently rated reason for discrimination for Hispanic/Latinx male veterans was race (42.50%) followed by ancestry (31.50%). For Hispanic/Latinx female veterans, it was gender (49.53%) followed by race (35.38%). For White male veterans, appearance (25.23%) and age (15.55%) were rated as the most frequent perceived reasons for discrimination, while gender (52.77%) and age (22.60%) were rated as the most frequent reasons for White female veterans. See Table 2 for a detailed comparison of EDS total mean score, EDS item means, and perceived reasons for discrimination by race/ethnicity and gender.

**Table 2:** EDS total score, EDS items, and EDS reasons by race/ethnicity and gender for entire sample and trauma-exposed sample.

**Entire Sample**														
	**White Male**		**White Female**		**Black Male**		**Black Female**		**Hispanic Male**		**Hispanic Female**			
	n = 978		n = 890		n = 284		n = 436		n = 202		n = 213			
**EDS total/item means**	**Mean**	**SD**	**Mean**	**SD**	**Mean**	**SD**	**Mean**	**SD**	**Mean**	**SD**	**Mean**	**SD**	**P value**	**R^2^**
EDS mean	9.65	9.09	9.21	8.14	13.13	11.28	12.14	10.09	11.18	10.67	10.67	8.78	< .0001	.020
EDS1: courtesy	1.51	1.52	1.54	1.40	1.86	1.63	1.90	1.61	1.66	1.58	1.73	1.53	< .0001	.010
EDS2: respect	1.46	1.49	1.54	1.40	1.74	1.64	1.82	1.61	1.60	1.58	1.66	1.53	.0006	.007
EDS3: Poor service	0.82	1.13	0.74	1.02	1.37	1.41	1.40	1.33	1.10	1.31	1.14	1.32	< .0001	.046
EDS4: Not smart	1.15	1.40	1.24	1.35	1.60	1.69	1.55	1.60	1.36	1.56	1.44	1.44	< .0001	.013
EDS5: Afraid of you	1.09	1.34	0.66	1.09	1.66	1.73	1.12	1.43	1.25	1.50	0.80	1.13	< .0001	.047
EDS6: Dishonest	0.65	1.09	0.46	0.92	1.11	1.47	0.83	1.32	0.90	1.38	0.62	1.06	< .0001	.029
EDS7: Better than	1.50	1.49	1.55	1.41	1.89	1.64	1.81	1.63	1.57	1.53	1.69	1.53	.0002	.008
EDS8: Insulted	0.96	1.26	0.86	1.15	1.16	1.51	1.08	1.40	0.98	1.37	0.94	1.24	.0067	.005
EDS9: Threatened	0.60	1.01	0.65	1.02	0.78	1.29	0.70	1.15	0.70	1.15	0.56	0.98	.0779	.003
**Reasons**	**N**	**%**	**N**	**%**	**N**	**%**	**N**	**%**	**N**	**%**	**N**	**%**	**p value**	**Cramer’s V**
Ancestry	48	4.94	26	2.94	50	17.99	71	16.32	63	31.50	45	21.23	< .0001	.286
Gender	122	12.56	467	52.77	68	24.46	231	53.10	22	11.00	105	49.53	< .0001	.405
Race	136	14.01	79	8.93	180	64.75	283	65.06	85	42.50	75	35.38	< .0001	.505
Age	151	15.55	200	22.60	57	20.50	90	20.69	47	23.50	49	23.11	.0021	.079
Religion	45	4.63	31	3.50	16	5.76	13	2.99	11	5.50	8	3.77	.3303	.044
Height	91	9.37	49	5.54	31	11.15	33	7.59	29	14.50	11	5.19	< .0001	.095
Weight	115	11.84	111	12.54	30	10.79	57	13.10	28	14.00	26	12.26	.8992	.023
Appearance	245	25.23	174	19.66	61	21.94	96	22.07	57	28.50	38	17.92	.0103	.071
Sexual Orientation	37	3.81	68	7.68	14	5.04	36	8.28	13	6.50	23	10.85	.0002	.089
**Trauma-Exposed Sample**														
	**White Male**		**White Female**		**Black Male**		**Black Female**		**Hispanic Male**		**Hispanic Female**			
	n = 599		n = 604		n = 183		n = 317		n = 125		n = 159			
**EDS total/item means**	**Mean**	**SD**	**Mean**	**SD**	**Mean**	**SD**	**Mean**	**SD**	**Mean**	**SD**	**Mean**	**SD**	**P value**	**R** ^**2**^
EDS mean	10.91	9.70	10.33	8.27	14.60	11.25	13.01	9.82	12.10	10.80	11.33	8.89	< .0001	.020
EDS1: courtesy	1.71	1.60	1.67	1.41	2.02	1.57	2.02	1.60	1.80	1.61	1.86	1.55	.0056	.008
EDS2: respect	1.66	1.56	1.69	1.40	1.82	1.58	1.95	1.61	1.72	1.59	1.79	1.58	.1219	.004
EDS3: Poor service	0.94	1.22	0.84	1.07	1.51	1.39	1.47	1.32	1.18	1.34	1.23	1.35	< .0001	.043
EDS4: Not smart	1.32	1.51	1.39	1.38	1.67	1.72	1.66	1.65	1.35	1.58	1.51	1.46	.0076	.008
EDS5: Afraid of you	1.25	1.44	0.78	1.15	1.86	1.73	1.23	1.41	1.45	1.60	0.88	1.17	< .0001	.053
EDS6: Dishonest	0.74	1.20	0.53	0.97	1.27	1.49	0.90	1.36	0.95	1.45	0.68	1.10	< .0001	.030
EDS7: Better than	1.67	1.56	1.68	1.43	2.12	1.61	1.93	1.65	1.67	1.57	1.65	1.54	.0028	.009
EDS8: Insulted	1.07	1.32	1.00	1.21	1.25	1.51	1.18	1.41	1.07	1.37	1.03	1.29	.2173	.004
EDS9: Threatened	0.69	1.09	0.74	1.05	0.93	1.34	0.80	1.18	0.81	1.20	0.60	1.01	.0575	.005
**Reasons**	**N**	**%**	**N**	**%**	**N**	**%**	**N**	**%**	**N**	**%**	**N**	**%**	**p value**	**Cramer’s V**
Ancestry	38	6.38%	20	3.32%	38	20.99%	57	18.04%	45	36.00%	36	22.64%	< .0001	.296
Gender	93	15.60%	335	55.56%	51	28.18%	177	56.01%	16	12.80%	77	48.43%	< .0001	.388
Race	94	15.77%	64	10.61%	127	70.17%	209	66.14%	57	45.60%	60	37.74%	< .0001	.508
Age	98	16.44%	144	23.88%	39	21.55%	60	18.99%	28	22.40%	35	22.01%	.0450	.076
Religion	38	6.38%	27	4.48%	12	6.63%	8	2.53%	9	7.20%	6	3.77%	.0920	.069
Height	61	10.23%	37	6.14%	21	11.60%	26	8.23%	18	14.40%	7	4.40%	.0033	.095
Weight	81	13.59%	90	14.93%	21	11.60%	49	15.51%	19	15.20%	18	11.32%	.6909	.039
Appearance	166	27.85%	133	22.06%	44	24.31%	75	23.73%	40	32.00%	32	20.13%	.0518	.075
Sexual Orientation	24	4.03%	58	9.62%	11	6.08%	27	8.54%	10	8.00%	20	12.58%	.0008	.103

*Note*. EDS = Everyday Discrimination Scale

Regarding differences between race/ethnicity groups in mental health symptoms, both Black veterans and Hispanic/Latinx veterans reported significantly more severe symptoms of PTSD, depression, and anxiety as compared to White veterans. Black veterans reported greater PTSD, depression, and anxiety symptoms as compared to Hispanic/Latinx veterans. However, Black veterans were significantly more likely to meet for probable PTSD (35.36%), depression (40.44%), and anxiety (36.73%) based on established clinical cut-offs as compared to both White (21.88%, 26.51%, and 23.29%, respectively) and Hispanic/Latinx veterans (27.85%, 33.42%, and 26.67%, respectively).

### Does EDS and income mediate the association between race/ethnicity and mental health symptoms?

A series of 12 models were run to examine whether EDS and/or income mediated the effect of race/ethnicity on mental health symptoms within each gender, controlling for age and education level. The bootstrap confidence intervals of these models are included in Table 3. Results support eight significant mediation models, which are described below.

**Table 3:** Bootstrap confidence intervals of direct and indirect effects in parallel mediation models.

Outcome	Gender	Race/Ethnicity Comparison	*R* ^2^	Total Direct Effect 95% CI	Total Indirect Effect 95% CI	EDS Indirect Effect 95% CI	Income Indirect Effect 95% CI
PHQ-9	Male	Black and White	.28	-1.57–0.26	1.48–2.73	0.45–1.40	0.85–1.54
PHQ-9	Male	Hispanic/Latinx and White	.26	-0.18–1.66	-0.01–1.10	-0.09–0.81	-0.06–0.43
PHQ-9	Female	Black and White	.22	-0.33–1.26	1.04–1.94	0.44–1.11	0.45–1.02
PHQ-9	Female	Hispanic/Latinx and White	.25	0.13–1.26	0.07–1.06	-0.19–0.63	0.13–0.60
GAD-7	Male	Black and White	.26	-1.09–0.53	1.14–2.19	0.44–1.29	0.53–1.09
GAD-7	Male	Hispanic/Latinx and White	.26	-0.26–1.35	-0.04–0.96	-0.12–0.73	-0.02–0.34
GAD-7	Female	Black and White	.20	-0.47–0.99	0.87–1.66	0.43–1.05	0.30–0.80
GAD-7	Female	Hispanic/Latinx and White	.20	-0.31–1.46	0.08–0.93	-0.07–0.68	0.06–0.40
PCL-5	Male	Black and White	.25	-1.69–5.51	3.79–7.93	1.09–4.39	1.95–4.46
PCL-5	Male	Hispanic/Latinx and White	.25	-0.29–7.05	-0.87–3.46	-0.91–2.68	-0.44–1.27
PCL-5	Female	Black and White	.19	-0.53–5.49	2.21–5.19	1.00–3.29	0.67–2.52
PCL-5	Female	Hispanic/Latinx and White	.20	-2.17–5.22	-0.31–3.13	-0.83–2.10	0.20–1.61

*Note*. EDS = Everyday Discrimination Scale; GAD-7 = Generalized Anxiety Disorder Questionnaire; PCL-5 = Posttraumatic Stress

Disorder Checklist for DSM-5; PHQ-9 = Patient Health Questionnaire

**Fig 1:**
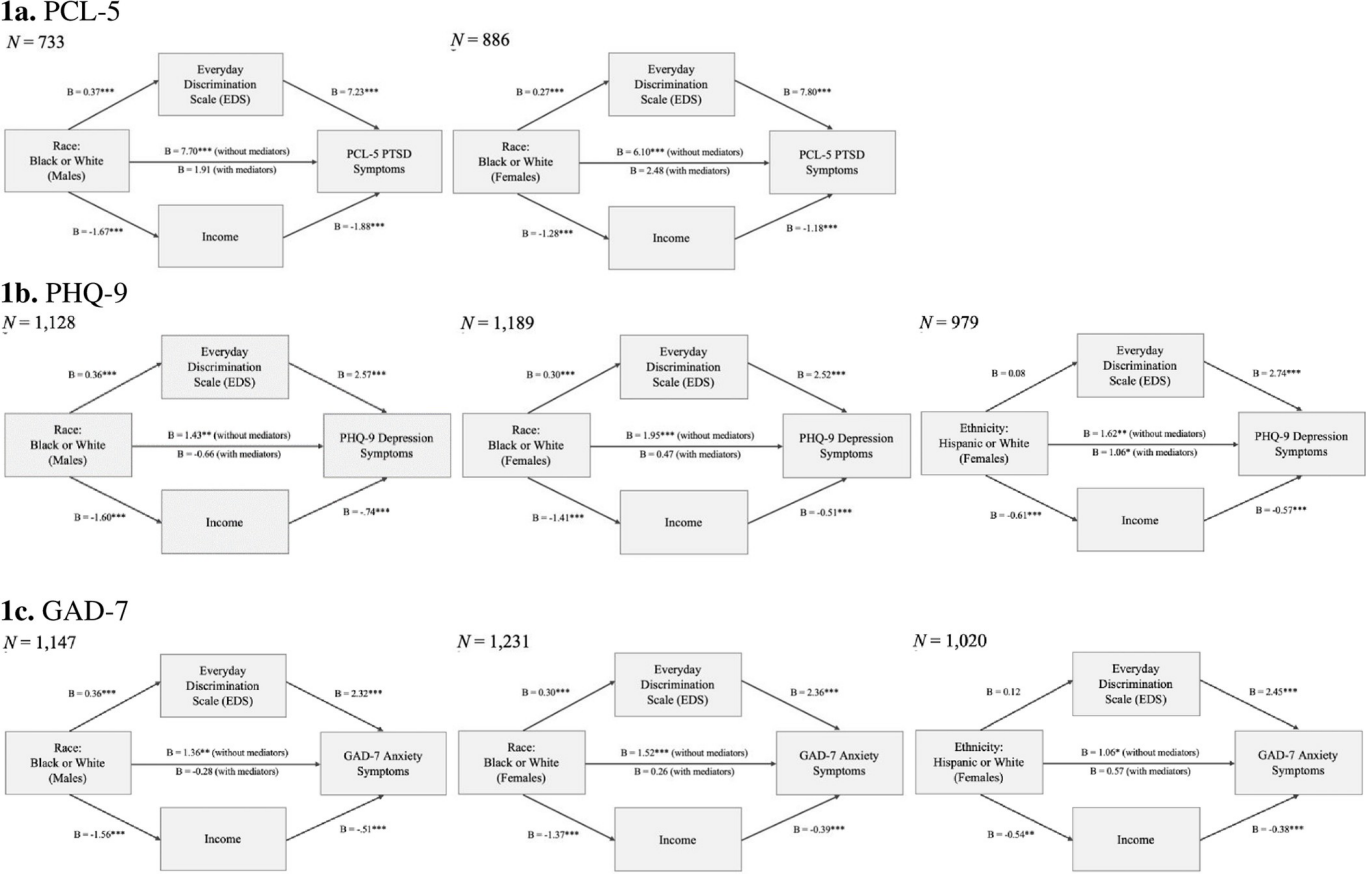
Mediating effects of everyday discrimination (EDS) and income on the association between race/ethnicity (Hispanic/Latinx vs. Non-Hispanic/Latinx White and Black vs. White) within male and female veterans on the PCL-5, PHQ-9, and GAD-7. a. PCL-5, b. PHQ-9, c. GAD-7. *Note*. *p < .05, **p < .01, ***p < .001; covariates: age, education.

For models comparing Black vs. White veterans the significant effect of race on PCL-5, PHQ-9, and GAD-7 was mediated by both EDS and income for both male and female veterans. Pairwise contrast testing revealed that EDS and income were not significantly different in strength as mediators across these models. Results varied in models comparing Hispanic/Latinx vs. White veterans. Hispanic/Latinx male vs. White male veterans showed no significant mediation effects of EDS or income on any of the mental health symptoms. However, Hispanic/Latinx female vs. White female veterans revealed that income, but not EDS, mediated the relationship between ethnicity and PHQ-9 and GAD-7. There were no mediation effects for Hispanic/Latinx female vs. White female veterans on the PCL-5. See Fig 1. Competing models that examined the mental health symptoms as the mediator revealed that in general the total indirect effect sizes were stronger in the original models, (i.e., everyday discrimination as the mediator), suggesting that everyday discrimination is more appropriate as the mediating variable between ethnoracial status and mental health.

## Discussion

Consistent with previous literature [10, 14, 20], this study found that both Black and Hispanic/Latinx veterans report more severe symptoms of PTSD, depression, and anxiety as compared to White veterans. Black veterans were also more likely to report sexual assault as the “worst event” with which they anchored their PTSD symptoms. For Black male and female veterans, both perceived everyday discrimination and income are mechanisms explaining a substantial portion of this increased risk after adjustment for age and education. These results align with the civilian literature, which have also found that both income/wealth [62] and discrimination [51, 63] partially explain racial/ethnic disparities in mental health symptoms. These findings also align with minority stress theory, suggesting that perceived everyday discrimination is an important mechanism through which marginalized social status negatively impacts mental health [25].

Our findings highlight the ways in which minoritized individuals bear a disproportionate burden of these social stressors. Although all groups reported perceived everyday discrimination, the sources of discrimination differ across groups. Our data suggests that perceived discrimination based on race has particularly insidious effects on mental health. Discrimination and low income are chronic stressors that may persist throughout a person’s lifespan [64]. Chronic experiences of discrimination and low income may impact the stress response system and increase allostatic load (i.e., harmful wear and tear on the body), which have subsequent long-term impacts on both mental and physical health [65, 66]. Furthermore, discrimination and low income are factors that not only impact an individual’s exposure to stress, but also the availability of structural resources (e.g. employment) necessary to mitigate the effects of that stress.

We did not observe gender differences in the models comparing Black vs. White participants, suggesting that both perceived everyday discrimination and income similarly explain the mental health disparity among both male and female Black veterans. Alternatively, results varied by gender when examining these mechanisms among Hispanic/Latinx veterans. Specifically, neither income nor perceived everyday discrimination mediated the association between race/ethnicity and mental health symptoms for male veterans. However, for Hispanic/Latinx female veterans, income explained a portion of the association for the increased risk of depression and anxiety, but not PTSD, symptoms. This suggests that the mechanisms explaining disparities between Hispanic/Latinx and White veterans in mental health symptoms varies by gender. These findings highlight the importance of examining disparities through an intersectional lens, as they illustrate that there is a unique experience of Hispanic/Latinx women as it relates to income that cannot be explained by gender or ethnicity alone. Hispanic/Latinx women may experience income-related stressors that are unique from those experienced by Hispanic/Latinx men and White women. As an example of the intersection of ethnicity and gender with respect to economic disparities, while the unemployment rate has narrowed between Hispanic/Latinx men and all other men (4.7% vs. 4.4% unemployed), the unemployment rate for Hispanic/Latinx women continues to remain higher compared to the rate for all other women (5.7% vs. 4.3%) [67]. Furthermore, Hispanic/Latinx women have shown the greatest increased rate of unemployment among all women [68]. Therefore, it is possible that income may be a more potent mechanism explaining disparities in mental health symptoms for Hispanic/Latinx women. In this sample, 22.54% of Hispanic/Latinx women had a household income of less than $25,000 compared with 12.31% of White women, while the difference between Hispanic/Latinx men and White men was more modest (15.81% vs. 11.42%). Furthermore, although Hispanic/Latinx Veterans experience significant differences in perceived everyday discrimination as compared to White veterans, this difference was modest as compared to the difference in perceived everyday discrimination between Black and White veterans. Therefore, perceived everyday discrimination may not be the most relevant mechanism explaining the disparity in mental health symptoms for Hispanic/Latinx veterans. Future research would benefit from further investigation of other potential mechanisms explaining mental health disparities for this subgroup.

This study was not without limitations. Unfortunately, there was not enough representation in other Non-Hispanic/Latinx racial groups (e.g., Native American) to allow for group comparisons. Second, the response rate in this study was relatively low (21%,) although consistent with other studies that have been conducted among veterans (i.e., response rates have ranged from 20%-30%) [69, 70], and there was some bias in response rate. This may impact the generalizability of the study findings. It is unknown to what extent veterans identified with their stated race and/or ethnicity, particularly among veterans that listed more than one race/ethnicity. Finally, there was variability in racial identities within the Hispanic/Latinx group, which may have impacted study findings and obscured the ability to see additional differences within this study subgroup. Unfortunately, we did not have enough veterans in the sample to create a White Hispanic/Latinx group and a Black Hispanic/Latinx group. Future research examining unique racial groups among Hispanic/Latinx identifying individuals would be important. Lastly, the cross-sectional nature of this study limits our ability to understand causality. However, competing models that switched the directionality of the mediation support the premise that everyday discrimination is one mediating factor explaining the association between ethnoracial status and mental health symptoms.

In sum, the current study provides further evidence of the role of perceived everyday discrimination and income in mediating racial/ethnic differences in mental health symptoms. Assessing and recognizing the potency of perceived discrimination is critical in informing our interventions for mental health disorders, particularly for vulnerable subgroups who report higher rates of perceived discrimination.
